# Transcriptional and translational‐uncoupling in regulation of the CXCL12 and its receptors CXCR4, 7 in THP‐1 monocytes and macrophages

**DOI:** 10.1002/iid3.199

**Published:** 2017-11-03

**Authors:** Lu Yu, Liangli Yu, Quynhchi Pham, Thomas T. Y. Wang

**Affiliations:** ^1^ Department of Nutrition and Food Science University of Maryland College Park Maryland 20742 USA; ^2^ USDA, Diet, Genomics and Immunology Laboratory Beltsville Human Nutrition Research Center, ARS Beltsville Maryland 20705 USA

**Keywords:** CXCL12, CXCR4, CXCR7, Lipopolysaccharide, THP‐1

## Abstract

**Introduction:**

The chemokine CXCL12 and its receptors CXCR4 and 7 play crucial roles in the immune system. In the present study, regulation of this pathway was further examined using the in‐vitro model of undifferentiated human THP‐1 monocytes (u‐THP‐1) and phorbol 12‐myristate 13‐acetate (PMA)‐differentiated THP‐1 macrophages (d‐THP‐1), to assess the effects of differentiation and the TLR4 ligand lipopolysaccharide (LPS) on the pathway.

**Methods/Results:**

Differentiation did not affect the CXCR4, 7 mRNA levels. Interestingly, the CXCL12 and CXCR7 proteins but not CXCR4 were found to be up‐regulated during differentiation. LPS, through CD14‐dependent pathway, induced CXCL12 and CXCR4, 7 mRNA levels to a greater magnitude in d‐ than u‐THP‐1. The induction effect on CXCL12 stimulated by LPS was confirmed using ELISA. Increased migration of u‐THP‐1 was observed using conditioned medium from LPS‐treated d‐THP‐1. Additionally, d‐THP‐1, although expressed higher CXCR7 protein levels, failed to migrate toward CXCL12. In contrast, LPS did not affect CXCR4, 7 protein levels.

**Conclusion:**

Hence, this study indicated that CXCL12, CXCR4, and CXCR7 were differentially expressed and regulated in u‐THP‐1 and d‐THP‐1 cells in response to external stimuli. Importantly, we reported here a novel observation that uncoupling exists between transcriptional and translational regulation of CXCR4, 7 expressions by differentiation and TLR stimuli.

## Introduction

Monocytes and macrophages respond to pathogens and provide a primary defense that ultimately lead to pathogen clearance 1, 2. During the pathogenic clearance process, differentiation of monocytes to macrophages is essential 3. Moreover, mounting immune responses in these cells often involve cross talk between cellular signaling pathways such as the pattern recognition and chemokine receptors‐mediated pathways 4. These cross‐talks are critical for the immune system to efficiently rid the body of pathogen, yet remain to be fully delineated. Chemokines are important signaling regulators of monocytes and macrophages trafficking, migration and thus, play an important role during immune responses 5. Chemokines are classified into four subgroups including CC, CXC, C, and CX_3_C, based on the position of cysteine residues 6. The chemokine CXC motif ligand 12 (CXCL12, or also known as stromal derived factor‐1, SDF‐1) is a member of CXC subgroup. CXCL12 not only modulates immune cell migration but may also be a critical contributor in tumor metastasis, organ development, wound healing, and angiogenesis 7, 8. CXCL12 is widely distributed in many types of tissues, organs and is continuously secreted by primary blood monocytes 7, 8, 9. CXCL12 acts via its two guanine nucleotide‐binding protein coupled CXC motif receptor (CXCR) 4 and 7 10. Binding of CXCL12 to CXCR4 is known to trigger the activation of CXCR4‐mediated downstream events that include, activation of matrix metalloproteinase‐9 enzyme, ERK signaling and the regulation of lymphocyte and phagocyte migration 11. CXCR7, on the other hand, is a newly discovered receptor for CXCL12 and was reported to serve as a decoy receptor for CXCL12 in some systems 12. The bio‐function and regulation of CXCR7 in monocytes and macrophages are not as well defined compared to those of CXCR4. Hence, further elucidation of the regulation and function of CXCR7 in monocytes and macrophages are warranted.

Recent studies have reported that exposure to lipopolysaccharides (LPS), a major outer membrane component of Gram‐negative bacteria and a Toll‐like receptor (TLR) ligand, can induce CXCL12 expression in THP‐1 monocytes (u‐THP‐1) at a pharmacological concentration (0.2 μg/mL) 13, 14. The Toll‐like receptors (TLRs) are a family of well‐known pattern recognition receptors in both monocytes and macrophages 15. The TLRs are known to induce inflammatory responses that converge at NF‐κB to stimulate the release of cytokines such as interleukin‐1 (IL‐1) and interleukin‐6 (IL‐6) 16, 17. Also, CXCR7 but not CXCR4 has been reported to be induced by LPS at high concentration (1 μg/mL) in combination with IFN‐γ, M‐CSF, or GM‐CSF 18. It is not known whether LPS alone, at physiological concentration (<100 ng/mL), can also elicit similar responses 14. However, these results suggest that a cross talk between TLR4 and the CXCL12/CXCR4, 7 pathway might exist. Moreover, LPS stimulation of inflammation through TLR4 involves interaction with two other proteins, CD14 and MD‐2 19, 20. LPS can act through both CD14‐dependent and independent pathways 21. The role of CD14 in the LPS‐mediated regulation of the CXCL12/CXCR4, 7 pathways has not been reported.

In addition to the inflammatory stimuli, monocyte differentiation has also been reported to regulate CXCL12/CXCR4, 7 pathways. Gupta et al. 22 observed up‐regulation of CXCR4 mRNA level within one hour of PMA induction of HL‐60 cell differentiation, but the mRNA levels declined to baseline level after three hours. Although mRNA expression of CXCR4 has been compared among human myeloid leukemia cell lines at different differentiation states in HL‐60, U‐937, THP‐1, and K‐562 cells, the expression of CXCL12 or CXCR7 was not reported in the study 22. Moreover, inconsistency in CXCR4 and 7 regulation exists in the literature. Contrary to Gupta et al., Ma et al. reported that, in differentiated THP‐1 (d‐THP‐1), CXCR7 but not CXCR4 was up‐regulated at mRNA and protein levels 18, 22. Hence, it is unclear whether coordinated changes in the CXCL12/CXCR4, 7 axis occurred during differentiation.

The critical nature of the CXCL12/CXCR4, 7 axis in immune responses, carcinogenesis, as well as a lack of full understanding of regulatory mechanism(s) for this pathway, prompted us to ask the following questions: (1) how do CXCL12/4, 7 respond to external stimuli such as LPS, differentiation, and whether CXCL12/CXCR4, 7 pathway in monocyte/macrophage is regulated in a coordinated manner; (2) at what concentration can LPS elicit a response in this pathway; and (3) what is the role of CD14 in LPS stimulated CXCL12/CXCR4, 7 response in monocyte/macrophage. We hypothesized that inflammatory and differentiation stimuli elicit coordinated regulation of CXCL12, CXCR4, and CXCR7 in monocyte and macrophage. The current study used the human THP‐1 cell monocyte/macrophage culture model to elucidate the effects of LPS and differentiation on the CXCL12/CXCR4, 7 pathways 23. Importantly, we reported here a novel observation that an uncoupling of transcriptional and translational responses of the CXCL12/CXCR4, 7 pathway occurred in monocyte and macrophage exposed to different stimuli.

## Results

### Comparison of CXCL12, CXCR4, 7 expression pattern in u‐THP‐1 and d‐THP‐1 cells

**Figure 1:**
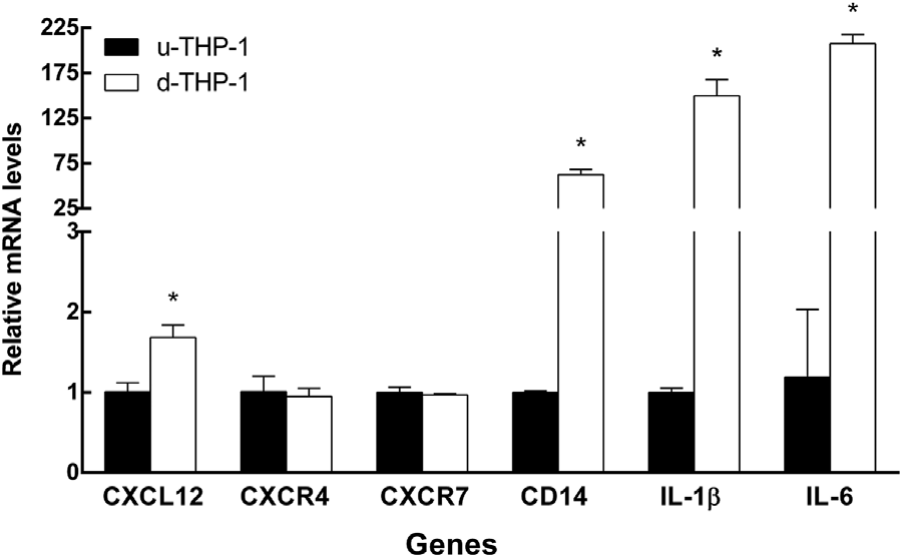
Comparison of CXCL12, CXCR4, CXCR7, CD14, IL‐1β, and IL‐6 mRNA levels in u‐THP‐1 and d‐THP‐1 without LPS induction. Undifferentiated (u‐THP‐1) and differentiated (d‐THP‐1) were cultured, total RNA isolated and gene expression of CXCL12, CXCR4, CXCR7, CD14, IL‐1β, and IL‐6 were determined using RT‐PCR as described in Materials and Methods. Results were normalized to u‐THP‐1 and expressed as relative mRNA levels (mean ± SD, *n* = 3). *p* values ≤ 0.05 were considered significant. * indicates significantly different from u‐THP‐1. Black bar, u‐THP‐1; White bar, d‐THP‐1.

**Figure 2:**
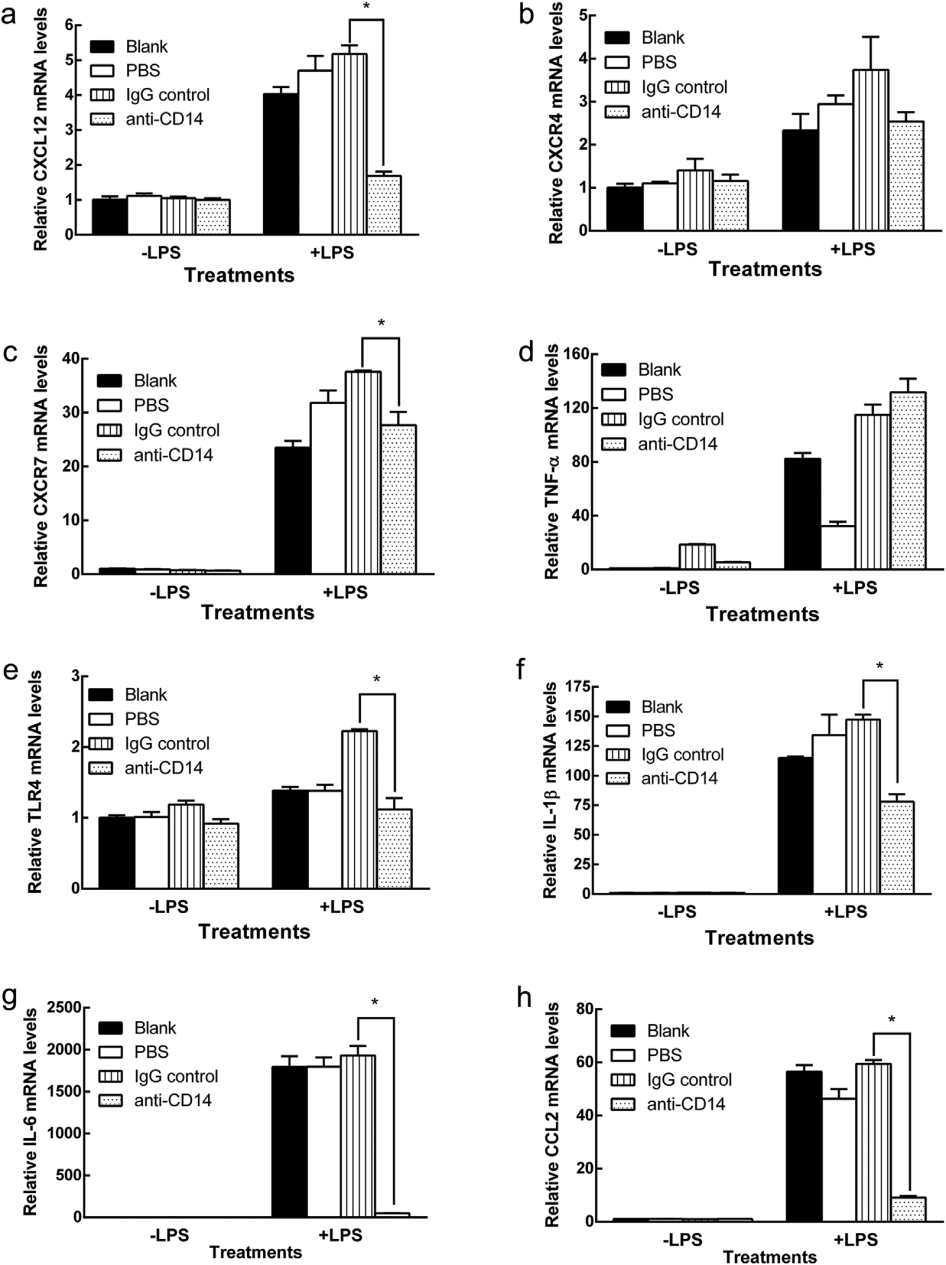
(a–g) Effects of anti‐CD14 antibody on LPS induction of gene expression in d‐THP‐1 cells. d‐THP‐1 were treated with mouse IgG isotype control or anti‐CD14 antibody (10 μg/mL) for one hour then medium was replaced with fresh medium with or without 10 ng/mL of LPS for an additional four hours. After LPS treatment, cells were harvested, total RNA isolated and mRNA levels of CXCL12, CXCR4, CXCR7, TNF‐α, TLR4, IL‐1β, IL‐6, and CCL2 were determined using RT‐PCR as described in Materials and Methods. Results were normalized to vehicle control and expressed as relative mRNA levels (mean ± SD, *n* = 3). *p* values ≤ 0.05 were considered significant and * indicates significantly different from control. (a) CXCR12. (b) CXCR4. (c) CXCR7. (d) TNF‐α. (e) TLR4 (f) IL‐1β. (g) IL‐6. (h) CCL2.

CXCL12 mRNA expressions in THP‐1 were significantly induced (1.68‐fold) after PMA‐induced differentiation (Fig. 1). In contrast, there was no difference in CXCR4 or CXCR7 mRNA levels between u‐THP‐1 and d‐THP‐1. In comparison, the differentiation marker CD14, cytokines IL‐1β and IL‐6 mRNA were up‐regulated in d‐THP‐1 by 62.48‐fold, 149.44‐fold, and 174.56‐fold, respectively (Fig. 1). LPS treatment of u‐THP‐1 or d‐THP‐1 significantly elevated CXCL12, IL‐1β, and IL‐6 mRNA levels (Fig. 1Sa‐c, Supplements). The magnitude of inductions on CXCL12 and IL‐6 mRNA expressions by LPS (10 ng/mL) was much greater in d‐THP‐1 than those in u‐THP‐1. In u‐THP‐1, CXCL12 and IL‐6 were increased by 2.97‐fold and 48.32‐fold, respectively. In d‐THP‐1, CXCL12 and IL‐6 were up‐regulated by 11.71‐fold and 150.91‐fold, respectively. Additionally, LPS induced CXCR7 mRNA level in both u‐THP‐1 and d‐THP‐1 cells by 1.81‐fold and 15.06‐fold, respectively. However, we only detected an increase in CXCR4 mRNA level in d‐THP‐1 but not in u‐THP‐1 upon LPS induction (Fig. 2Sa‐b, Supplements).

### Characterization of LPS‐induced CXCL12/CXCR4, 7 and related gene expression in d‐THP‐1 cells

**Figure 3:**
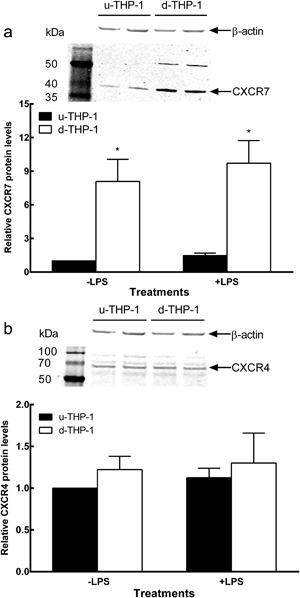
(a–b) Effects of LPS on CXCR4, 7 protein levels in u‐THP‐1 and d‐THP‐1. Cells were cultured and treated with or without LPS. Cells were harvested for protein determination and western blot as described in Materials and Methods. Immunoreactive bands were quantitated using *ODYSSEY*® CLx Infrared Imaging System as described in Materials and Methods. Results were normalized to vehicle control and expressed as relative protein levels (mean ± SD, *n* = 3). *p* values ≤ 0.05 were considered significant and * indicates significantly different from control. The panel represents (a) β‐actin and CXCR7. (b) β‐actin and CXCR4.

**Figure 4:**
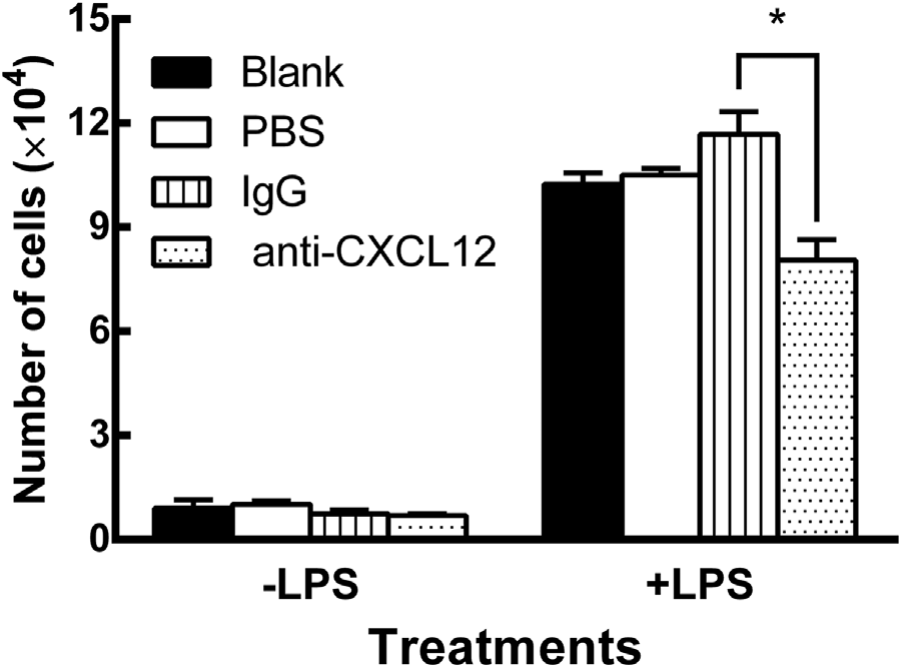
Effect of LPS and anti‐CXCL12 antibody on u‐THP‐1 migration towards conditioned media. THP‐1 migration towards conditioned media harvested from d‐THP‐1 treated with or without LPS (10 ng/mL) was conducted as described in Materials and Methods. Cells migrated to the bottom wells were stained with Trypan Blue and counted under the microscope. For experiment using the anti‐CXCL12 antibody, the conditioned media were pre‐treated with IgG isotype control or anti‐CXCL12 antibody for one hour prior to initiation of migration. Results were normalized to vehicle control and expressed as mean ± SD (*n* = 3). *p* values ≤ 0.05 were considered significant and * indicates significantly different from control.

Given that LPS elicits a more robust response in d‐THP‐1, the time and concentration‐dependent effects of LPS on CXCL12, and CXCR4, 7 mRNAs were further characterized in d‐THP‐1 cells. LPS induced CXCL12 mRNA expression in a dose‐ and time‐dependent manner. CXCL12 mRNA level peaked at 4, 4, 10, and 12 hours with 1, 10, 25, and 100 ng/mL of LPS, respectively (Fig 4Sa, Supplements). CXCL12 mRNA declined almost to the baseline 24 hours after addition of LPS. Interestingly, CXCL12 showed distinct LPS induction patterns compared to CXCR4, 7 (Fig. 3Sb‐c, Supplements). CXCR4, 7 mRNA levels both peaked at two hours after LPS induction, and the magnitude of CXCR7 induction was much higher compared to CXCR4. CXCR7 mRNA increased by as much as 12.4‐fold following two hours of LPS induction (25 ng/mL), while CXCR4 mRNA only elevated by 2.6‐fold in the same condition. Additionally, unlike CXCL12, the expression of CXCR4, 7 mRNA leveled off at 25 ng/mL LPS.

As a comparison, LPS significantly up‐regulated relative mRNA levels of IL‐1β and IL‐6, two well documented LPS‐responsive cytokines in d‐THP‐1 24. The expressions peaked at 4 h (Fig. 4Sa‐b, Supplements). Both IL‐1β and IL‐6 showed time‐ and concentration‐dependent up‐regulation by LPS.

### Blocking antibody against CD14 inhibited CXCL12‐related gene expression in LPS induced d‐THP‐1

LPS acts through both CD14‐dependent and CD14‐independent pathways 21. The role of CD14 in response to LPS in the regulation of CXCL12 and its receptors were tested. After one hour pre‐treatment using human CD14 blocking antibody, LPS induction of CXCL12 mRNA level was effectively reduced by 78.7% when compared to the IgG control (Fig. 2a). In contrast, the effect of CD14 blocking antibody on CXCR7 inhibition was relatively lower than that on CXCL12. LPS induction of CXCR7 was inhibited by 26.4%, compared to the IgG control (Fig. 2b and c). CD14‐independent gene, TNF‐α, was not affected by CD14 blocking antibody (Fig. 2d). At two hours treatment of LPS (10 ng/mL), TLR4 mRNA level was reduced by 49.8% compared to IgG control (Fig. 2e). Consistently, mRNA expressions of the known downstream genes of TLR4 including IL‐1β, IL‐6, and CCL2 were all attenuated by CD14 blocking antibody. IL‐1β mRNA was reduced by 47.0% while IL‐6 and CCL2 mRNA reduced by 97.5% and 97.0% respectively as compared to the IgG control (Fig. 2f–h).

### LPS induced CXCL12 protein secretion in THP‐1 cells

Human CXCL12 protein level was determined by ELISA using media collected from d‐THP‐1 treated with LPS (10 ng/mL) for 24 hours. CXCL12 levels remained low in media for the first 12 hours, but its secretion significantly increased (6.73‐fold, *p* < 0.001) following 24 hours of LPS treatment (Fig. 5Sa, Supplements). Comparing u‐THP‐1 and d‐THP‐1, significantly elevated CXCL12 protein level was observed in media from d‐THP‐1 (*p* < 0.001) after 24 hours exposure to LPS, but not from u‐THP‐1 (Fig. 5Sb, Supplements).

### Effect of differentiation and LPS on CXCR4, 7 protein levels in THP‐1 cells

Effect of differentiation and LPS on CXCR4, 7 proteins were examined using Western blot. Differentiation significantly increased CXCR7 protein (Fig. 3a) level, but not CXCR4 protein (Fig. 3b) level. On the other hand, unlike the results at the mRNA level, no difference was observed in CXCR4, 7 protein levels when cells were treated with LPS (Fig. 3a and b).

### CXCL12 neutralizing antibody blocked u‐THP‐1 migration toward d‐THP‐1‐conditioned media

Migration of u‐THP‐1 toward d‐THP‐1‐conditioned media in the presence or absence of a neutralizing antibody to CXCL12 was used as a functional assay to validate the presence of CXCL12 (Fig. 4). The media collected from two hours LPS (10 ng/mL) treated d‐THP‐1 without CXCL12 antibody accelerated u‐THP‐1 cell migration by 11.3 to 12.9‐fold compared to that without LPS treatment. Treatment of conditioned media with CXCL12 neutralizing antibody but not IgG control significant inhibited LPS‐induced migratory effect (Fig. 4).

### Comparison of migration of u‐and d‐THP‐1 towards CXCL12

The ability of cells to migrate toward CXCL12 was compared between u‐THP‐1 and d‐THP‐1. In the absence of CXCL12, a chemoattractant, the percentage of d‐THP‐1 cells migrated to the bottom well was about half of that of u‐THP‐1 (Fig. 6S, Supplements). In the presence of CXCL12 (10 ng/mL), the percentage of u‐THP‐1 migrated to the bottom well was significantly increased (2.05‐fold, *p *< 0.001). In contrast, no difference in migration was detected in d‐THP‐1 with or without CXCL12 treatment (Fig. 6S, Supplements).

## Discussion

The current study addresses several deficiencies in the literature regarding the regulation of CXCL12 and its receptors in monocyte/macrophages. We provide mechanistic insights into the regulation of the CXCL12/CXCR4, 7‐pathway through detailed analyses of responses of the CXCL12/CXCR4, 7 pathway toward differentiation and a TLR4 stimulus, LPS. We also provide data supporting the role of CD14 in LPS‐induced response through the pathway. More importantly, as summarized in Table 1, we report here a novel observation that an uncoupling between transcriptional and translational machinery exists in the regulation of CXCL12/CXCR4, 7 pathway by different stimuli.

**Table 1:** The effects of (1) THP‐1 differentiation and (2) LPS stimulation on expressions of CXCL12 and CXCR4, 7 in THP‐1 cells at mRNA and protein level

	Genes		
Expressions	CXCL12	CXCR4	CXCR7
1. Effect of differentiation on gene expressions			
mRNA	↑	–	–
Protein	↑	–	↑
2. Effect of LPS on gene expressions			
a. u‐THP‐1			
mRNA	↑	–	↑
Protein	–	–	–
b. d‐THP‐1			
mRNA	↑	↑	↑
Protein	↑	–	–

In this study, we found differentiation of monocyte to macrophage had no effect on CXCR4 and CXCR7 at mRNA level (Fig. 1). In contrast, the cytokines CXCL12, IL‐1β, and IL‐6 mRNA (Fig. 1) were significantly increased upon differentiation. Interestingly, despite the lack of difference in CXCR7 mRNA level, at the protein level, both CXCL12 (Fig. 5S, Supplements) and CXCR7 proteins (Fig. 4) were significantly increased in d‐THP‐1 compared to u‐THP‐1. Protein levels sometimes might not be correlated with mRNA levels, especially in higher organisms 25, 26. Post‐transcriptional regulation of gene expression, mRNA decay, translation, protein half‐lives and protein degradation play crucial roles in the determination of steady state protein concentrations 25, 27. The observation of this study supports that, during differentiation, uncoupling exists between mRNA and protein expressions of CXCR7. The lack of CXCR4 response to PMA at the mRNA level, observed in this study, is different from a previous report in which Gupta et al. reported an increase of CXCR4 mRNA expression during HL‐60 monocyte differentiation within one hour after the stimulation of GM‐CSF 22. This may be partially explained by the different cell types, inducing agent, and length of treatment used in the two studies, which warrant further elucidation.

The transcriptional and translational uncoupling was also observed in the responses of THP‐1 cells to the TLR4 stimulus, LPS. At mRNA level, induction with LPS for four hours significantly increased both CXCR4, 7 in d‐THP‐1 when compared to that in u‐THP‐1. However, neither CXCR4 nor 7 proteins changed upon exposure to LPS (Fig. 3). In contrast, CXCL12 mRNA, as well as protein, was induced by LPS, which is consistent with a recent report 13, supporting inflammatory effect elicited by bacteria is in part mediated through regulation of CXCL12 but not CXCR4, 7. Moreover, we observed that LPS at a physiologically relevant 20‐fold lower concentration (10 ng/mL) 13, 14 than that reported by Hung et al., elicited a response through CXCL12. These data further support a physiological role for CXCL12 in immune response towards bacteria in monocytes/macrophages. The responses of the CXCL12/CXCR4, 7 pathway toward inflammatory stimuli also differ between monocytes and macrophages. Treatment with LPS elicits stronger responses in d‐THP‐1 cells than those in u‐THP‐1 cells for CXCL12, CXCR4, 7 at mRNA level, similar to the effect of LPS on other well documented LPS‐responsive genes, such as IL‐1β and IL‐6. Our results show that LPS induction of CXCL12 and CXCR4, 7 mRNAs is CD14 dependent, as pre‐treatment of CD14 blocking antibody inhibited LPS induced increase of CXCL12, CXCR7 mRNA expressions (Fig. 2). Also, CD14 was up‐regulated during differentiation (Fig. 1). Hence, this enhanced transcriptional response may be in part due to the increase in CD14 27. Interestingly, CD14 blocking antibody appeared to differentially block LPS induction of downstream genes. CXCL12, IL‐6, and CCL2 expressions appeared to be inhibited to a greater extent than those of CXCR4, 7, or IL‐1β. These results suggest that CXCR4, 7, and IL‐1β mRNA may be also regulated by CD14 independent pathway. Additional studies are warranted to dissect these differences.

Conditioned media from the LPS induction of d‐THP‐1 significantly increased the migratory effect of u‐THP‐1 (Fig. 4), which is consistent with a previous observation by Gouwy et al. 28. However, in our study the protein level of CXCL12 after LPS stimulation reached around 0.1 ng/mL in the media, this concentration is much lower than 1 ng/mL reported by Gouwy et al. It was also observed in this study that neutralizing CXCL12 in conditioned media using CXCL12 antibody partially inhibited u‐THP‐1 migration by 31.0% when compared to the IgG control (Fig. 4). This is consistent with LPS induction of other cytokines such as CCL5, CCL3, and CCL8 that synergize with CXCL12 to affect monocyte migration but support the fact that CXCL12 also plays a crucial role in promoting monocyte migration 28.

CXCL12 is a relatively ubiquitously expressed chemokine 29. As shown in our conditioned media migration assay, production of CXCL12 by macrophage will further attract monocytes. Monocyte, which has lower expression level of CXCR7, will migrate to the inflamed site and enhance the immune response. Given the proposed role of CXCR7 as a decoy receptor 12, the higher CXCR7 protein levels in combination with no change in CXCR4 protein levels in differentiated macrophage may help the macrophage reside at the inflamed local site instead of migrating toward CXCL12 produced by other tissues. Our comparison of u‐THP‐1 and d‐THP‐1 migration supported this notion. We observed that u‐THP‐1 and not d‐THP‐1 migrated toward CXCL12 (Fig. 6S, Supplements). Hence, we consider that CXCR7 serves as a decoy receptor in the macrophage which binds to CXCL12 and short circuit the CXCL12/CXCR4 interaction.

Activation of CXCL12/CXCR4 not only is involved in the migration of monocyte but also the migration of T cell 30, dendritic cell 31, neuronal cell as well as cancer cell 32. For example, migration of prostate cancer cell could be induced by CXCL12/CXCR4 activation through Akt‐1 and MMP‐9 signaling pathway and migration of pancreatic cancer cells is modulated through MMP‐2 and MMP‐9 pathway 33, 34. The chemotactic effect on multiple cell lines by CXCL12 and its receptors might indicate the global importance of CXCL12/CXCR4, 7 chemokine axis in the modulation of cell migration and mediates cross‐talk as well as the interaction between immune and cancer cells. More importantly, the role of CXCR7 needs to be further defined. Based on our results as well as others’, we reason that CXCR7 serves as a decoy to CXCR4 and can potentially nullify CXCL12's biological effects including migration. Human peripheral blood monocytes are known to contain multiple cell sub‐types 35, 36 and differentiation signal such as those induced by PMA also can lead to generation of multiple cell types 37, 38. It remains unclear how the CXCL12/CXCR4,7 pathways are regulated among the cells. Understanding the precise role of CXCR7 in other cell types would be critical in future studies.

In summary, the CXCL12/CXCR4, 7‐axis in THP‐1 cells is subject to multiple regulations including differentiation and TLR4 ligand. The CXCL12/CXCR4, 7 chemokine axis is differentially regulated when exposed to differentiation agent and TLR‐mediated inflammatory stimuli. Additionally, we observed the uncoupling of CXCR4, 7 expressions at the transcriptional and translational level. Regulation at post‐transcriptional level appeared to contribute to changes in CXCR7. Also, CXCL12 appeared to work in concert with other chemokines in the regulation of monocyte migration. Finally, our results also support a decoy role for CXCR7 in monocyte/macrophage. Overall these changes may facilitate and direct cell migration, localization during inflammation.

## Materials and Methods

### Ethical statement

An ethical statement is not required as there were no human subjects involved in this study.

### Materials and reagents

Human monocytic leukemia cell line THP‐1 was purchased from American Type Culture Collection (Manassas, VA). Phorbol 12‐myristate 13‐acetate (PMA) and lipopolysaccharides from *Escherichia coli* 0111:B4 were obtained from Sigma–Aldrich (St Louis, MO). Penicillin and streptomycin (pen‐strep), RPMI medium with phenol red, fetal bovine serum, TRIzol reagent, TaqMan Fast Universal PCR master mix, primers for Tbp (Hs00427620_m1), CXCL12 (Hs00171022_m1), CXCR4 (Hs00237052_m1), CXCR7 (Hs00604567_m1), TLR4 (Hs00152939_m1), TNF‐α (Hs00174128_m1), CCL2 (Hs00234140_m1), IL‐1β (Hs01555410_m1), IL‐6 (Hs00985639_m1), NuPAGE antioxidant, NuPAGE LDS sample buffer, NuPAGE reducing agent, iBlot gel transfer stacks, and NuPAGE MES SDS running buffer were all purchased from Life Technologies (Grand Island, NY). The 8 μm pore polycarbonate membrane inserts 24‐well plates, Pierce RIPA buffer, Halt protease inhibitor cocktail, 20X TBS buffer, 20X TBS tween‐20 buffer were from Thermo Fisher Scientific (Waltham, MA). Mouse IgG1 isotype control, human CD14 blocking antibody (Cat #MAB3832), human CXCL12 neutralizing antibody (Cat #AF‐310‐NA) and human CXCL12/SDF‐1α Immunoassay ELISA kit (Cat #DSA00) were purchased from R&D Systems Inc (Minneapolis, MN). Mouse monoclonal β‐actin antibody (Cat #sc‐47778) and rabbit polyclonal anti‐CXCR4 antibody (Cat #sc‐9046) were from Santa Cruz Biotechnology (Dallas, TX). Rabbit monoclonal anti‐GPCR RDC1 (CXCR7) (Cat #ab138509) antibody was purchased from Abcam (Cambridge, MA). IRDye 800 CW goat anti‐mouse (Cat #925‐32210) and goat anti‐rabbit (Cat #925‐32211) secondary antibody were obtained from LI‐COR Biosciences (Lincoln, NE).

### Cell culture

Human u‐THP‐1 monocytes were maintained in RPMI medium (RPMI 1640 with glutamine and phenol red, 10% FBS, 1% pen‐strep). The d‐THP‐1 were obtained after 48 hours exposure in the presence of the differentiation agent PMA (25 ng/mL) in RPMI medium.

### Effect of LPS and differentiation on gene expression

To compare CXCL12, CXCR4, and CXCR7 mRNA levels in u‐THP‐1, cells were plated in RPMI medium at the density of 2.5 × 10^5^ cells/mL in six‐well plates at 37°C in 5% CO_2_. Treatment initiated after 24 hours. For d‐THP‐1, 5 × 10^5^ cells/mL of human u‐THP‐1 were seeded in six‐well plate in RPMI medium in the presence of differentiation agent PMA (25 ng/mL). After 48 hours‐treatment with PMA, cells attached to the bottom of the plate and treatments were initiated. For u‐THP‐1 and d‐THP‐1 comparison studies, LPS treatment was for four hours at 10 ng/mL then the cells were collected for RNA isolation and gene expression determination. For concentration and time course experiment with d‐THP‐1, LPS at 0, 1, 10, 25, 100 ng/mL were added to the cells after 48 h incubation in PMA. Concentrations of LPS were chosen based on effective ranges described in previous work 14. D‐THP‐1 cells were harvested at 0, 2, 4, 8, 10, 12, 16, 20, 24 hours respectively for RNA isolation and gene expression determination.

### Effect of CD14 blocking antibody on d‐THP‐1 cell's responses to LPS

The effects of CD14 blocking antibody on CXCL12 expression was studied using the anti‐human CD14 mAb (Clone # 134620). D‐THP‐1 cells on six‐well plate were obtained as described above. Mouse IgG control and CD14 antibody (10 μg/mL) were added to the cells for one hour. The medium was then replaced with or without 10 ng/mL of LPS and incubated for four hours before the cells were collected for RNA isolation and gene expression analysis.

### RNA isolation, cDNA synthesis and Real‐time PCR analysis of gene expression

RNA isolation, cDNA synthesis, and Real‐time PCR analysis of gene expression were performed as described previously 39. Briefly, total RNA were isolated using the TRIzol reagent (Life Technologies, Carlsbad, CA) and Affinity Script Multiple Temperature cDNA Synthesis kit (Agilent Technologies, Santa Clara, CA) was used to reverse transcribe mRNA to cDNA. Real‐time PCR was performed on ViiA7 Real‐Time PCR Detection System using the TaqMan Universal Fast Master Mix and TaqMan gene expression assay (Life Technologies, Carlsbad, CA) to quantify gene expression levels of human CXCL12, CXCR4, 7, IL‐1β, and IL‐6, and CCL2. Human TATA box binding protein (TBP) was used as a housekeeping gene for calculation of relative expression levels using the ddCt method as previously described 39.

### Determination of CXCL12 production using ELISA

The d‐THP‐1 were obtained by culturing in RPMI medium in the presence of PMA for 48 hours in six‐well plates as described above. LPS (10 ng/mL) was added and media (100 μL) were collected every four hours during the 24 hours treatment. For u‐THP‐1 and d‐THP‐1 comparison study, cells were cultured in six‐well plate as described above and media (100 μL) were collected from both u‐THP‐1 and d‐THP‐1 treated with/without 10 ng/mL of LPS for 24 hours. CXCL12 protein levels in the media were determined using a commercially available human CXCL12/SDF‐1α ELISA kit according to the manufacturer's instructions (R&D Systems, Minneapolis, MN).

### Western blot analysis of CXCR4, 7 protein levels

The protein levels of CXCR4 and CXCR7 in u‐THP‐1 and d‐THP‐1 with/without LPS treatments were assessed using Western blot analysis. The u‐THP‐1 and d‐THP‐1 cells (5 × 10^5^ cells/mL) were treated with/without 10 ng/mL of LPS for 2, 4, and 24 hours in T‐175 cm^2^ flasks and were harvested. For u‐THP‐1, cells were washed once with 15 mL of phosphate‐buffered saline (PBS) and centrifuged at 1500 rpm for 5 minutes. After centrifugation, PBS was removed and cells were lysed in 200 µL RIPA buffer containing EDTA and protease inhibitors. The lysates were homogenized on ice three times (10 s each) using a Branson digital sonifier (Branson, CT), samples were then centrifuged at 10,000 rpm for 15 min at 4°C. The supernatant was collected and protein concentrations were tested using BCA assay following manufacturer's protocol (Thermo Scientific, Rockford, IL). For d‐THP‐1, cells were washed with 15 mL of PBS and scrapped off using cell Falcon scraper (Corning Life Sciences, Acton, MA). The harvested cells were then centrifuged at 1500 rpm for 5 minutes and 200 µL RIPA lysis buffer containing EDTA and protease inhibitors was added to cells after PBS removal. The lysates were homogenized three times on ice (10 s each), using a Branson digital sonifier (Branson, CT), samples were then centrifuged at 10,000 rpm for 15 min at 4°C. The supernatant was collected and protein concentration was determined using the Pierce BCA assay following manufacturer's protocol (Thermo Scientific, Rockford, IL). Routinely, 5 μg per sample was used for electrophoresis separation on SDS–PAGE using 10% Bis‐Tris gel following manufacturer's protocol (NUPAGE, Invitrogen, Carlsbad, CA). After electrophoresis, the protein was transferred from the gel onto a nitrocellulose membrane using iBlot apparatus according to manufacturer's procedure (Invitrogen, Carlsbad, CA). After the transfer, nitrocellulose membrane was then blocked in 1× TBS buffer with 5% milk for one hour at room temperature while shaking, followed by washing with SuperBlock T20 (TBS) blocking buffer (three times, 5 min each). After washing, the membrane was then incubated with loading control mouse monoclonal β‐actin antibody (1:1000), primary antibody rabbit polyclonal anti‐CXCR4 (1:2000) or rabbit monoclonal anti‐GPCR RDC1/CXCR7 (1:10000) in blocking solution overnight at 4°C. After overnight incubation, the membrane was washed with 1X TBS Tween 20 buffer (three times, 5 min each) and incubated with IRDye 800 CW goat anti‐mouse secondary antibody (1:20000 for β‐actin), and IRDye 800 CW goat anti‐rabbit secondary antibody (1:10000 for CXCR4 and 1:20000 for CXCR7) in SuperBlock T20 for two hours, in the dark, at room temperature. After secondary antibody incubation, the membrane was washed using 1X TBS Tween 20 buffer and preserved in 1X TBS buffer before imaging and quantitation. Specific CXCR4 and CXCR7 proteins were detected and quantitated using the LICOR ODYSSEY® CLx Infrared Imager according to manufacturer's procedure (LiCOR, Lincoln, NE).

### Cell migration assay

The migration of THP‐1 cells toward CXCL12 as a functional assay was assessed according to a published method 40. An 8 μm pore polycarbonate membrane insert (Thermo Fisher Scientific Inc, Pittsburgh, PA) in 24‐well plates was used for the chemotaxis assay. For conditioned media experiment, d‐THP‐1 cell medium collected after 24 hours treatment of LPS induction (10 ng/mL) was used as conditioned medium. Conditioned media (400 μL) were added to the bottom wells of the 24‐well chemotaxis chambers. The u‐THP–1 (200 μL, 1 × 10^6^ cells/mL) were added to the upper chamber and incubated at 37°C in 5% CO_2_. After five hours, the insert was removed, stained with Trypan Blue and the cells migrated to the bottom wells were counted under microscopy (10× magnification) using hemocytometer. Experiments where CXCL12 neutralizing antibody was used to assess the specific effect of CXCL12 on migration 40, conditioned media collected were treated with/without a neutralizing CXCL12 antibody for one hour before being added to the lower bottom wells, and numbers of cells migrated after five hours were counted as described above. To compare u‐THP‐1 and d‐THP‐1 migration ability, u‐THP‐1 (200 μL, 1 × 10^6^ cells/mL) were added to the upper chamber, CXCL12 containing media (10 ng/mL) was placed at the bottom well and incubated at 37°C/5% CO_2_. After five hours, the insert was removed, stained with Trypan Blue and the cells migrated to the bottom wells were counted under the microscope (10× magnification) using hemocytometer. For d‐THP‐1 cell migration/chemotaxis assay, 24‐well plates with 8 μm pore polycarbonate membrane inserts (Thermo Fisher Scientific Inc, Pittsburgh, PA) were used. THP‐1 monocytes (200 μL of 1 × 10^6^ cells/mL) with 25 ng/mL PMA in RPMI medium were added to the upper inserts and 400 μL of RPMI medium were added to the bottom wells of the 24‐well chemotaxis chambers. After 48 hours differentiation at 37°C/5% CO_2_, media in the upper inserts were changed to fresh media and media at the bottom were replaced with CXCL12 containing media (10 ng/mL). After five hours, cells in the upper inserts were removed using cotton swabs for the counting of migrated cells. For the total cell number, cells in upper inserts were not removed by swabbing. Cells were stained with crystal violet (0.2% in 20% ethanol). After 20 minutes of staining, inserts were rinsed three times using DI water, and then plates were placed in the hood to be air dried. Finally, crystal violet was dissolved in 10% acetic acid (500 μL) and the absorbance of the solution was read at 560 nm using the Molecular Devices SPECTRAMAX 384Plus (Sunnyvale, CA).

### Statistical analysis

All experiments were performed in triplicate, and data were reported as the mean ± standard deviation (SD). GraphPad Prism for Windows (Prism 4, GraphPad Software Inc., La Jolla, CA) was used for statistical analysis. Depending on the experimental design, multiple group experiments were analyzed using one‐ or two‐way ANOVA followed by post‐hoc test. *p* values ≤ 0.05 were considered significant.

## Acknowledgments

This work was supported by United States Department of Agriculture (USDA) appropriated fund #8040‐51530‐056‐00D. L.Y. and T.T.W. conceived, designed the experiments and wrote the paper. L.Y. performed experiments, analyzed the data and prepared the figures. Q. P. performed experiments. All authors discussed the results and commented on the manuscript. The authors also thank Dr. H. Huang for his editorial assistance.

## Conflicts of Interest

The authors declare no commercial or financial conflict of interest.

## Supporting information

Additional supporting information may be found in the online version of this article at the publisher's web‐site.

**Figure 1Sa‐c**. Comparison of LPS induced CXCL12, IL‐1β and IL‐6 expression in u‐THP‐1 and d‐THP‐1.
**Figure 2Sa‐b**. Comparison of LPS induction of CXCR4, 7 expression in u‐THP‐1 and d‐THP‐1.
**Figure 3Sa‐c**. Concentration‐ and time‐dependent effects of LPS on CXCL12/CXCR4, 7 expression in d‐THP‐1 cells.
**Figure 4Sa‐b**. Effects of LPS on IL‐1β and IL‐6 expression in d‐THP‐1 cells.
**Figure 5Sa‐b**. Effects of LPS on CXCL12 protein in THP‐1 cells
**Figure 6S**. Migration of u‐THP‐1 and d‐THP‐1 towards CXCL12 (10 ng/mL).
