# Supplementary material for: Transcriptional and translational‐uncoupling in regulation of the CXCL12 and its receptors CXCR4, 7 in THP‐1 monocytes and macrophages
Source: Immun Inflamm Dis. 2017 Nov 3;6(1):106–16. doi: 10.1002/iid3.199 (PMC5818454; doi:10.1002/iid3.199)
Supplement: Supplementary file 1 — Figure 1Sa‐c. Comparison of LPS induced CXCL12, IL‐1β and IL‐6 expression in u‐THP‐1 and d‐THP‐1. Figure 2Sa‐b. Comparison of LPS induction of CXCR4, 7 expression in u‐THP‐1 and d‐THP‐1. Figure 3Sa‐c. Concentration‐ and time‐dependent effects of LPS on CXCL12/CXCR4, 7 expression in d‐THP‐1 cells. Figure 4Sa‐b. Effects of LPS on IL‐1β and IL‐6 expression in d‐THP‐1 cells. Figure 5Sa‐b. Effects of LPS on CXCL12 protein in THP‐1 cells Figure 6S. Migration of u‐THP‐1 and d‐THP‐1 towards CXCL12 (10 ng/mL). [file IID3-6-106-s001.docx]

**Supporting Information**

**Figure Legends**

**Figure 1Sa-c.** **Comparison of LPS induced CXCL12, IL-1β and IL-6 expression in u-THP-1 and d-THP-1.** u-THP-1 (2.5 × 10^5^ cells/mL) and d-THP-1 (5 × 10^5^ cells/mL) treated with 10 ng/mL of LPS for 2 h, total RNA isolated and mRNA levels of CXCL12, IL-1β, and IL-6 were determined using RT-PCR as described in Materials and Methods. Results were normalized to vehicle control and expressed as relative mRNA levels (mean ± SD, *n* = 3). *P* values ≤ 0.05 were considered significant and * indicates significantly different from control. (a) CXCL12. (b) IL-1β. (c) IL-6. Black bar: u-THP-1, White bar: d-THP-1.

**Figure 2Sa-b.** **Comparison of LPS induction of CXCR4, 7 expression in u-THP-1 and d-THP-1.** u-THP-1 (2.5 × 10^5^ cells/mL) and d-THP-1 (5 × 10^5^ cells/mL) treated with 10 ng/mL of LPS for 2 h, total RNA isolated and mRNA levels of CXCL4 and 7 were determined using RT-PCR as described in Materials and Methods. Results were normalized to vehicle control and expressed as relative mRNA levels (mean ± SD, *n* = 3). *P* values ≤ 0.05 were considered significant and * indicates significantly different from control. (a) CXCR4. (b) CXCR7. Black bar: u-THP-1, White bar: d-THP-1.

**Figure 3Sa-c. Concentration- and time-dependent effects of LPS on CXCL12/CXCR4, 7 expression in d-THP-1 cells.** d-THP-1 were treated with 0, 1, 10, 25 and 100 ng/mL of LPS and harvested at 0, 2, 4, 8, 10, 12, 16, 20 and 24 h. Total RNA isolated and mRNA levels for CXCL12, CXCR4, CXCR7 were determined using RT-PCR as described in Materials and Methods. Results were normalized to 0 h and expressed as relative mRNA levels (mean ± SD, *n* = 3). *P* values ≤ 0.05 were considered significant and * indicates significantly different from 0 ng/mL of LPS treatment. (a) CXCL12. (b) CXCR4. (c) CXCR7.

**Figure 4Sa-b. Effects of LPS on IL-1β and IL-6 expression in d-THP-1 cells.** d-THP-1 were treated with 0, 1, 10, 25 and 100 ng/mL of LPS and harvested at 0, 2, 4, 8, 10, 12, 16, 20 and 24 h. Total RNA isolated and mRNA levels of IL-1β and IL-6 were determined using RT-PCR as described in Materials and Methods. Results were normalized to 0 h and expressed as relative mRNA levels (mean ± SD, *n* = 3). P values ≤ 0.05 were considered significant and * indicates significantly different from 0 ng/mL of LPS treatment. (a) IL-1β. (b) IL-6.

**Figure 5Sa-b. Effects of LPS on CXCL12 protein in THP-1 cells.** (a) Time-dependent effect of LPS on CXCL12 protein in d-THP-1. Data were reported as mean CXCL12 protein (pg/mL) ± SEM, n = 3. *P* values ≤ 0.05 were considered significant and * indicates significantly different from 0 h. (b) Comparison of LPS effect on CXCL12 protein in u-THP-1 and d-THP-1. U-THP-1 or d-THP-1 cells were cultured and treated with LPS (10 ng/mL). Medium harvested after 24 h and CXCL12 protein were determined using ELISA as described in the Materials and Methods. Data were reported as mean CXCL12 protein (pg/mL) ± SD, *n* = 3. *P* values ≤ 0.05 were considered significant and * indicates significantly different from vehicle control.

**Figure 6S.** **Migration of u-THP-1 and d-THP-1 towards CXCL12 (10 ng/mL).** uTHP-1 and d-THP-1 migration towards CXCL12 (10 ng/mL) were conducted as described in Materials and Methods. u-THP-1 cells migrated to the bottom wells (with 10 ng/mL of CXCL12) were stained with Trypan Blue after 5 h of migration and counted under the microscope. For d-THP-1, monocytes were treated with PMA (25 ng/mL) for 48 h. After cell differentiation, migration of d-THP-1 towards 10 ng/mL of CXCL12 (5 h) was tested. For migrated cell counting, cells in the upper inserts were removed using cotton swabs. For total cell counting, cells in upper inserts were not removed by swabbing. Crystal violet-stained cells were rinsed, dried and re-dissolved in 10% acetic acid. Absorbance was read at 560 nm. Results were normalized to vehicle control and expressed as mean ± SD (*n* = 3). *P* values ≤ 0.05 were considered significant and * indicates significantly different from control.

**Figures**

**Figure 1Sa-c.** **Comparison of LPS induced CXCL12, IL-1β and IL-6 expression in u-THP-1 and d-THP-1.** (a) CXCL12. (b) IL-1β. (c) IL-6.





**Figure 2Sa-b. Comparison of LPS induction of CXCR4, 7 expression in u-THP-1 and d-THP-1.** (a) CXCR4. (b) CXCR7.





**Figure 3Sa-c. Concentration- and time-dependent effects of LPS on CXCL12 and CXCR4, 7 expression in d-THP-1 cells.** (a) CXCL12. (b) CXCR4. (c) CXCR7.





**Figure 4Sa-b. Effects of LPS on IL-1β and IL-6 expression in d-THP-1 cells.** (a) IL-1β. (b) IL-6.





**Figure 5Sa-b. Effects of LPS on CXCL12 protein in THP-1 cells.** (a) Time-dependent effect of LPS on CXCL12 protein in d-THP-1. (b) Comparison of LPS effect on CXCL12 protein in u-THP-1 and d-THP-1.





**Figure 6S.** **Migration of u-THP-1 and d-THP-1 towards CXCL12 (10 ng/mL).**
